# MGAT5 regulates ferroptosis via the Nrf2/HO-1 signaling pathway in diabetic nephropathy

**DOI:** 10.1515/biol-2025-1362

**Published:** 2026-09-08

**Authors:** Lina Wang, Xiangjun Meng, Meng Wang, Lingbo Guan

**Affiliations:** Department of Endocrinology, Linping Campus, The Second Affiliated Hospital of Zhejiang University School of Medicine, Hangzhou, Zhejiang, China; Department of Intensive Care Unit, Linping Campus, The Second Affiliated Hospital of Zhejiang University School of Medicine, Hangzhou, Zhejiang, China

**Keywords:** diabetic nephropathy, nuclear factor erythroid 2-related factor 2/heme oxygenase-1, mannosyl (alpha-1,6-)-glycoprotein beta-1,6-N-acetylglucosaminyltransferase, ferroptosis, inflammatory responses

## Abstract

Diabetic nephropathy (DN) is a major complication of diabetes mellitus, and identifying effective therapeutic targets remains an urgent need. In this study, genes associated with DN were obtained from the gene expression omnibus (GEO) database, and Bioinformatics analysis revealed that mannosyl (alpha-1,6-)-glycoprotein beta-1,6-N-acetylglucosaminyltransferase (MGAT5) was downregulated in DN. Functional experiments demonstrated that MGAT5 overexpression activated the nuclear factor erythroid 2-related factor 2 (Nrf2)/heme oxygenase-1 (HO-1) pathway, enhanced DN-related cell viability, and reduced the levels of lactate dehydrogenase (LDH), cleaved caspase-3 (C-casp3), Bcl-2-associated X protein (Bax), interleukin-6 (IL-6), interleukin-1 beta (IL-1β), tumor necrosis factor-alpha (TNF-α), malondialdehyde (MDA), acyl-CoA synthetase long-chain family member 4 (ACSL4), prostaglandin-endoperoxide synthase 2/cyclooxygenase-2 (PTGS2/COX-2), Fe^2+^ content, ROS production, and apoptosis. Meanwhile, MGAT5 overexpression increased the levels of B-cell lymphoma 2 (Bcl-2), glutathione (GSH), and glutathione peroxidase 4 (GPX4). Conversely, MGAT5 knockdown partially reversed these protective effects. These findings suggest that MGAT5 may alleviate DN-associated cellular injury by activating the Nrf2/HO-1 pathway and regulating oxidative stress, inflammatory responses, ferroptosis, and apoptosis. MGAT5 may represent a potential therapeutic candidate for further investigation in diabetic nephropathy.

## 1 Introduction

Diabetic nephropathy (DN) represented a prevalent chronic consequence of diabetes [1], 2], characterized by persistent proteinuria, gradually declining glomerular filtration rate, and often accompanied by typical diabetic glomerular lesions [3]. The development of DN was influenced by multiple factors, such as obesity, hyperglycemia, and genetic predisposition [4], [5], [6], [7]. With improvements in living standards, the prevalence of obesity increased, and the number of diabetic patients continued to rise; approximately 20–40 % of diabetic patients eventually developed DN, resulting in a growing population of DN patients worldwide [8], [9], [10]. DN progressed from microalbuminuria to overt proteinuria, leading to impaired renal function and ultimately end-stage renal disease [11], 12], and it was often associated with risks such as hypertension, atherosclerosis, and stroke [13], [14], [15], which significantly reduced patients’ quality of life and even increased mortality [16], 17].

Currently, the treatment of DN focuses on slowing disease progression, protecting renal function, and reducing complications. Some commonly prescribed agents, including inhibitors of sodium-glucose cotransporter 2 (SGLT2) [18], blockers of the angiotensin-converting enzyme (ACE) [19], and antagonists of angiotensin II receptors (ARB) [20], help mitigate the malignant progression of DN. However, the mechanisms of DN are very complex, and it remains difficult to fully reverse the condition. Many studies showed that the nuclear factor erythroid 2-related factor 2/heme oxygenase-1 (Nrf2/HO-1) signaling pathway functioned as a crucial mediator in DN progression [21], [22], [23], modulating oxidative stress, inflammation, and apoptosis in renal cells, making it a potential therapeutic target for slowing DN progression [24], [25], [26]. Nevertheless, the upstream regulators and downstream effects of this pathway in DN remain unclear, and new molecular targets are still being explored to advance precision therapy.

Protein glycosylation is an essential post-translational modification that regulates protein stability, cellular localization, signal transduction, and biological functions. N-acetylglucosaminyltransferase V (MGAT5), a key glycosyltransferase involved in the branching modification of N-glycans, plays important roles in various cellular processes, including inflammation, cell adhesion, and stress responses [27], 28]. Increasing evidence has indicated that abnormal glycosylation patterns are closely associated with the development of kidney diseases and may influence renal cell injury by regulating oxidative stress and inflammatory responses [29], 30]. In addition, emerging studies have suggested that glycosylation-related pathways may interact with ferroptosis-related processes, which are increasingly recognized as important mechanisms involved in diabetic kidney injury [31], 32]. However, the role of MGAT5 in DN progression and its potential regulatory relationship with the Nrf2/HO-1 signaling pathway remain largely unknown.

Therefore, investigating the molecular mechanisms of DN, especially the key regulators affecting the Nrf2/HO-1 pathway, is of great significance for revealing critical steps in DN development and uncovering potential avenues for therapeutic intervention. In the present study, MGAT5 was identified through bioinformatics analysis and selected for further investigation based on its potential involvement in glycosylation-mediated cellular stress regulation. This work is designed to investigate the upstream regulators and downstream consequences of the Nrf2/HO-1 pathway in DN and to further assess their effects on renal cell function, thereby offering a theoretical and experimental foundation for precision intervention in DN.

## 2 Materials and methods

### 2.1 Dataset download and differentially expressed genes (DEGs) screening

The DN-related dataset GSE30122 was obtained from the gene expression omnibus (GEO, https://www.ncbi.nlm.nih.gov/geo/) repository, and the dataset included transcriptome information from 69 kidney tissue samples of controls and DN patients. The expression data were normalized according to standard preprocessing procedures, and probe annotation was performed based on the corresponding microarray platform.

Differential expression analysis was performed using the R package “limma”. Linear model fitting, contrast matrix construction, and empirical Bayes moderation were applied, and DEGs were extracted using the “topTable” function. The Benjamini-Hochberg method was used to control the false discovery rate. Genes with adjusted *P* value (adj*P*) < 0.05 and |log_2_ fold change (|log_2_FC|) ≥ 2 were considered significantly differentially expressed [33], [34], [35], resulting in 117 DEGs, including 12 upregulated and 105 downregulated genes. GO and KEGG enrichment analyses were performed to investigate the biological functions and signaling pathways associated with DEGs. Literature review indicated that MGAT5, an N-acetylglucosaminyltransferase, was involved in glycosylation modification [28], 36], a process that significantly contributed to DN development [37]. Therefore, MGAT5 was selected for further investigation.

### 2.2 Cell culture

Human podocyte cells (HPC) were purchased from Huatuobio Co., Ltd. (Shenzhen, China). Frozen HPC were taken out of liquid nitrogen and rapidly warmed in a 37 °C water bath for about 3 min. Cells were then diluted with prewarmed 89 % McCoy’s 5A medium (Huatuobio) supplemented with 10 % fetal bovine serum (FBS, Procell system, Wuhan, China) and 1 % penicillin-streptomycin (P/S, Huatuobio) and spun down to remove the cryoprotectant. The resulting pellet was resuspended in fresh medium, evenly distributed into culture flasks, and maintained at 37 °C under 5 % CO_2_. The medium was refreshed every 2–3 days, and cell morphology was observed to confirm they remained in the logarithmic growth phase. When cultures reached roughly 80 % confluence, cells were treated with 0.1 % trypsin (Procell system), gently separated, and subcultured. HPC cells between passages 3 and 8 were used for all experiments.

The cells were assigned to two experimental groups: the first, the normal glucose (NG) group, was maintained in medium containing 5.5 mM glucose (Bioss, Beijing, China) along with 24.5 mM mannitol (Yitabio, Beijing, China) to serve as an osmotic control; the second, the high glucose (HG) group, was exposed to medium supplemented with 30 mM glucose (Bioss) to mimic an HG environment and establish a DN cell model. The total osmolarity between the NG and HG groups was precisely matched by supplementing mannitol in the NG group. The concentration of 30 mM glucose was selected to simulate a hyperglycemic condition and induce DN-related cellular injury in HPC cells. Cells were exposed to HG conditions for 48 h before subsequent experiments. Following this, an additional HG+MGAT5+ML385 group was prepared according to experimental design. In this group, 5 µM ML385 (Bioss) was applied during MGAT5 transfection to investigate the interaction between Nrf2/HO-1 and MGAT5.

### 2.3 Cell transfection

HPC cells in the logarithmic growth phase under HG conditions were seeded into 96-well plates. For MGAT5 overexpression experiments, cells were transfected with the pcDNA3.1-MGAT5 plasmid (HG+MGAT5, GenePharma, Shanghai, China), while cells transfected with the empty pcDNA3.1-vector served as the control group (HG+vector, GenePharma). For MGAT5 knockdown experiments, cells were transfected with MGAT5-specific small interfering RNA (si-MGAT5, GenePharma), while non-targeting siRNA (si-NC, GenePharma) was used as the negative control. All transfections were performed using Lipofectamine 3000 reagent (Invitrogen, Carlsbad, California, USA) according to the manufacturer’s instructions.

The efficiency of MGAT5 overexpression and knockdown was evaluated by detecting MGAT5 expression levels using RT-qPCR and western blot analysis.

### 2.4 Western blot (WB)

Total protein levels were measured using a bicinchoninic acid (BCA) Protein Assay Kit (Elabscience, Wuhan, China). Proteins with equal quantities were resolved by sodium dodecyl sulfate-polyacrylamide gel electrophoresis (SDS-PAGE) and subsequently transferred onto polyvinylidene fluoride (PVDF) membranes (Elabscience), which were then blocked with 5 % bovine serum albumin (BSA) (Elabscience) for 2 h at room temperature.

The membranes were subsequently incubated overnight at 4 °C with primary antibodies, including β-actin (ab8226, 1:500, Abcam, Cambridge, UK), MGAT5 (ab87977, 1:500, Abcam), cleaved caspase-3 (C-casp3) (ab2302, 1:500, Abcam), Bcl-2-associated X protein (Bax) (ab182733, 1:500, Abcam), B-cell lymphoma 2 (Bcl2) (ab32124, 1:500, Abcam), glutathione peroxidase 4 (GPX4) (ab41787, 1:500, Abcam), acyl-CoA synthetase long-chain family member 4 (ACSL4) (ab205197, 1:500, Abcam), prostaglandin-endoperoxide synthase 2 (PTGS2) (ab300668, 1:500, Abcam), nuclear factor erythroid 2-related factor 2 (Nrf2) (ab137550, 1:500, Abcam), and heme oxygenase-1 (HO-1) (ab85309, 1:500, Abcam). After washing, membranes were exposed to HRP-linked secondary antibodies (ab6789 and ab97051, 1:2,000, Abcam) for 1 h at room temperature. Protein signals were then detected with an enhanced chemiluminescence (ECL) kit (Elabscience), and the band intensities were quantified using ImageJ software (v2.0).

### 2.5 Reverse transcription quantitative polymerase chain reaction (RT-qPCR)

Cells in the logarithmic growth phase from each group were disrupted using TRIzol reagent (Invitrogen, Waltham, Massachusetts, USA), and total RNA was subsequently isolated according to the manufacturer’s guidelines. A quantity of one μg of total RNA was then subjected to reverse transcription into cDNA using the PrimeScript RT Kit (Takara, Kusatsu, Japan) following the provided protocol. For gene expression analysis, RT-qPCR was conducted with SYBR Green qPCR Master Mix (Takara), using glyceraldehyde-3-phosphate dehydrogenase (GAPDH) as the internal control, and the relative levels of the target gene MGAT5 were determined via the 2^−ΔΔCt^ calculation method. All reactions were performed in triplicate, and the primer sequences are detailed in Table 1.

**Table 1:** RT-qPCR primer sequence.

Gene	Forward (5′-3′)	Reverse (5′-3′)
MGAT5	AAA​TTG​AGG​ACG​GAC​TCC​CG	GGA​CGG​TCT​GCA​AAC​TAC​CA
β-actin	CTG​GAA​CGG​TGA​AGG​TGA​CA	GTC​CTC​GGC​CAC​ATT​GTG​AA

### 2.6 Cell counting kit-8 (CCK8)

Cells in the logarithmic growth phase were resuspended at a density of 8 × 10^3^ cells/well and seeded uniformly into 96-well plates, followed by incubation for 24 h to allow cell attachment. Subsequently, 10 µL of CCK-8 solution was added to each well and gently mixed, and the plates were incubated at 37 °C with 5 % CO_2_ for 3 h. The absorbance at 450 nm was measured using a microplate reader (Olympus, Tokyo, Japan). Each group was analyzed with at least three independent replicates. Wells containing only culture medium and CCK-8 solution were used as blank controls. The CCK-8 assay was used to evaluate relative cellular metabolic activity, which indirectly reflected changes in cell viability.

### 2.7 Measurement of lactate dehydrogenase (LDH), Fe^2+^, malondialdehyde (MDA), glutathione (GSH) levels

LDH assay kits (Elabscience), Fe^2+^ assay kits (Elabscience), malondialdehyde (MDA) assay kits (Biogradetech, Waltham, MA, USA), and glutathione (GSH) assay kits (Biogradetech) were used to measure the levels of LDH, Fe^2+^, MDA, and GSH in each group of cells, following the manufacturers’ protocols strictly. Finally, the optical density (OD) of the samples was measured with a NanoDrop 2000 UV–Vis spectrophotometer (Thermo Scientific, Waltham, Massachusetts, USA).

### 2.8 Flow cytometry

After treatment, cells were collected and washed twice with phosphate-buffered saline (PBS, pH = 7.4; Elabscience). Cell apoptosis was assessed by staining with an annexin V-fluorescein isothiocyanate (FITC)/propidium iodide (PI) apoptosis detection kit (Elabscience) according to the manufacturer’s instructions. The cells were incubated in the dark for 15 min during staining. After staining, the cells were washed with PBS, resuspended, and immediately analyzed using a BD FACSCanto™ II flow cytometer (BD Biosciences, Franklin Lakes, New Jersey, USA). The acquired data were analyzed using FlowJo 10.2 software to evaluate the level of apoptosis.

For reactive oxygen species (ROS) detection, treated cells were washed with PBS (Elabscience), followed by incubation with the fluorescent probe provided in the ROS detection kit (Wanleibio) according to the manufacturer’s instructions. The cells were incubated at 37 °C in the dark. After staining, intracellular ROS levels were detected using a BD FACSCanto™ II flow cytometer (BD Biosciences). The data were analyzed using FlowJo 10.2 software.

For lipid ROS detection, treated cells were washed with PBS (Elabscience), followed by staining with the C11-BODIPY 581/591 fluorescent probe (1:1,000; Wanleibio) according to the instructions of the lipid ROS detection kit (Wanleibio). The cells were incubated at 37 °C in the dark. After staining, intracellular lipid ROS levels were detected using a BD FACSCanto™ II flow cytometer (BD Biosciences). The data were analyzed using FlowJo 10.2 software.

### 2.9 Enzyme-linked immunosorbent assay (ELISA) detection

The interleukin-6 (IL-6) kit (BPS Bioscience, San Diego, California, USA), interleukin-1 beta (IL-1β) kit (BPS Bioscience), and tumor necrosis factor-alpha (TNF-α) kit (MultiSciences, Hangzhou, China) were used to measure the corresponding inflammatory and oxidative stress markers in the collected cell samples. Each assay was performed carefully following the guidelines provided by the manufacturers, and the concentrations of each target molecule were quantified by measuring absorbance values using a microplate reader (Tiangen, Beijing, China).

### 2.10 Statistical analysis

Each experimental procedure was executed in a minimum of three independent iterations. Quantitative data were delineated as the mean ± standard deviation (SD). Statistical evaluations were carried out employing GraphPad Prism 8.0 software, whereupon Gaussian distribution of the datasets was authenticated via the Shapiro-Wilk normativity test. Divergences between dual cohorts were scrutinized through Student’s *t*-test, whereas variance among multiple cohorts was adjudicated via analysis of variance (ANOVA), further substantiated by Tukey’s post-hoc test for multiple comparisons. A threshold of *P* < 0.05 was deemed statistically meaningful.

## 3 Results

### 3.1 MGAT5 expression is decreased in tissues of DN patients

**Figure 1:**
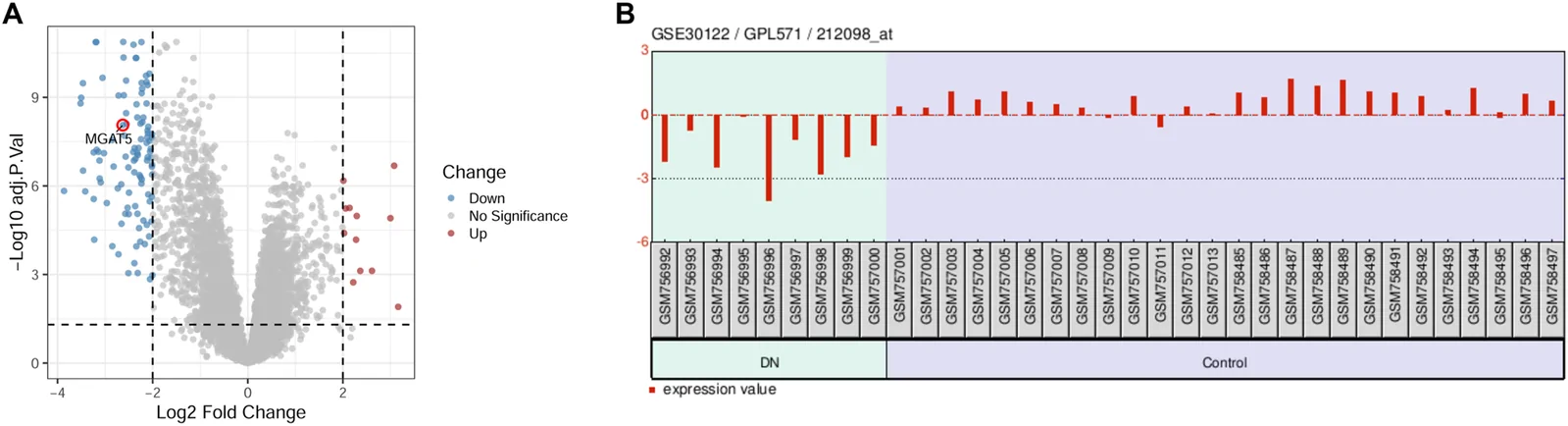
MGAT5 expression in DN. (A) Volcano plot of DN-related DEGs based on the GEO database, with red indicating upregulation and blue indicating downregulation; *P*adj < 0.05 indicates significant differences. (B) Expression of MGAT5 across samples in the DN-associated GSE30122 dataset based on the GEO database (control = 26, DN = 9).

Data analysis revealed that MGAT5 expression was reduced in tissues from patients with DN (Figure 1A and B). This suggests that the downregulation of MGAT5 may play an important role in the pathological process of DN.

### 3.2 HG suppresses MGAT5 expression in HPC cells

**Figure 2:**
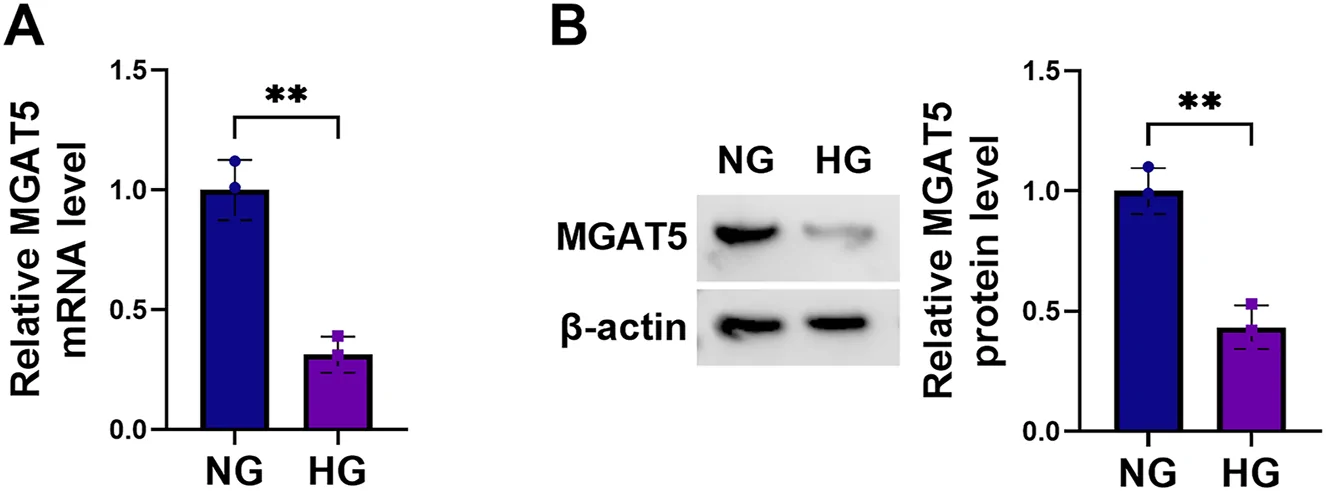
Validation of MGAT5 expression in DN cell models. (A) mRNA expression levels of MGAT5 in DN cell models measured by RT-qPCR (*n* = 3). (B) Protein expression levels of MGAT5 in DN cell models measured by WB (*n* = 3). **P < 0.01.

An *in vitro* DN model was established using HPC cells exposed to high glucose, and both RT-qPCR and WB analyses showed a marked reduction in MGAT5 mRNA and protein levels (Figure 2A and B). These findings indicate that MGAT5 downregulation may represent an important molecular feature of high glucose-induced pathological alterations in HPC cells.

### 3.3 MGAT5 overexpression alleviates HG-induced apoptosis and inflammation in HPC cells

**Figure 3:**
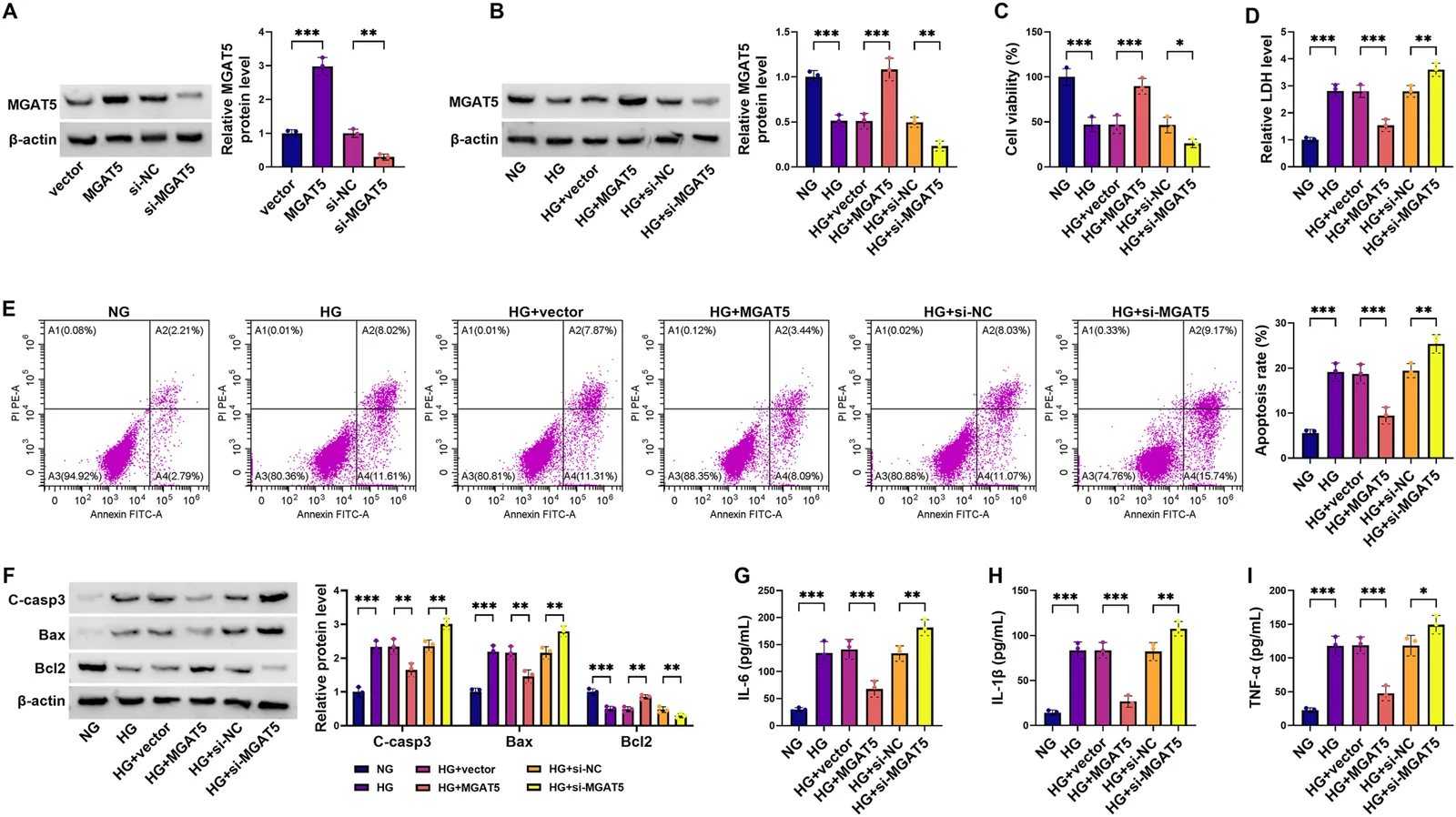
Effects of MGAT5 on the function of DN cell models. (A) Validation of MGAT5 overexpression and knockdown efficiency in normal cells by WB (*n* = 3). (B) Validation of MGAT5 overexpression and knockdown efficiency in DN cell models by WB (*n* = 3). (C) Effects of MGAT5 overexpression and knockdown on cell viability in DN cell models by CCK-8 (*n* = 3). (D) Effects of MGAT5 overexpression and knockdown on LDH levels in DN cell models by colorimetric assay (*n* = 3). (E) Effects of MGAT5 overexpression and knockdown on apoptosis in DN cell models by flow cytometry (*n* = 3). (F) Effects of MGAT5 overexpression and knockdown on C-casp3, Bax, and Bcl2 protein expression in DN cell models by WB (*n* = 3). (G) Effects of MGAT5 overexpression and knockdown on IL-6 levels in DN cell models by ELISA (*n* = 3). (H) Effects of MGAT5 overexpression and knockdown on IL-1β levels in DN cell models by ELISA (*n* = 3). (I) Effects of MGAT5 overexpression and knockdown on TNF-α levels in DN cell models by ELISA (*n* = 3). *P < 0.05, **P < 0.01, ***P < 0.001.

To verify the regulatory role of MGAT5 in DN, this study overexpressed MGAT5 and investigated its effects on the biological functions of DN cell models. WB analysis confirmed that MGAT5 was successfully overexpressed and silenced under both normal conditions and following NG or HG treatment (Figure 3A and B). Under HG stimulation, MGAT5 overexpression markedly enhanced cell viability, reduced LDH release and apoptosis, and was associated with decreased levels of C-casp3 and Bax along with increased Bcl2 expression, but the opposite trends were observed under HG treatment alone and with MGAT5 knockdown (Figure 3C–F). Moreover, MGAT5 overexpression lowered the levels of pro-inflammatory cytokines IL-6, IL-1β, and TNF-α, whereas MGAT5 silencing exerted the opposite effects, but the opposite trends were observed under HG treatment alone as well as with MGAT5 knockdown (Figure 3G–I). These results indicate that MGAT5 plays a protective role against HG-induced injury in HPCs by enhancing cell viability and suppressing apoptosis and inflammation.

### 3.4 MGAT5 overexpression mitigates HG-induced oxidative stress and ferroptosis in HPC cells

**Figure 4:**
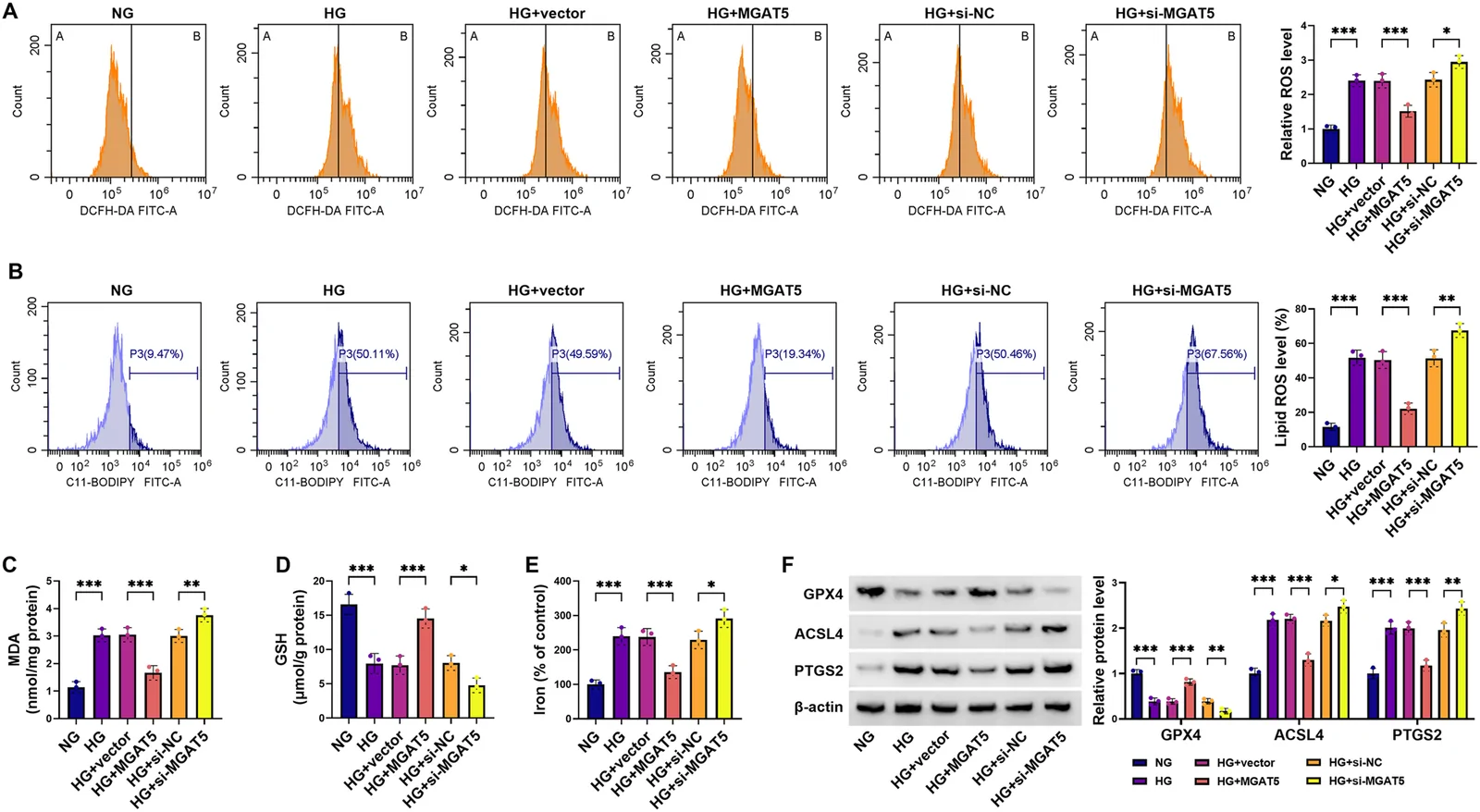
Effects of MGAT5 on oxidative stress and ferroptosis in DN cell models. (A) Effects of MGAT5 overexpression and knockdown on ROS levels in DN cell models measured by flow cytometry (*n* = 3). (B) Effect of MGAT5 overexpression and knockdown on lipid ROS levels in the DN cell model detected by flow cytometry (*n* = 3). (C) Effects of MGAT5 overexpression and knockdown on MDA levels in DN cell models measured by colorimetric assay (*n* = 3). (D) Effects of MGAT5 overexpression and knockdown on GSH levels in DN cell models measured by colorimetric assay (*n* = 3). (E) Effects of MGAT5 overexpression and knockdown on Fe^2+^ levels in DN cell models measured by colorimetric assay (*n* = 3). (F) Effects of MGAT5 overexpression and knockdown on protein expression levels of GPX4, ACSL4, and PTGS2 in DN cell models measured by WB (*n* = 3). *P < 0.05, **P < 0.01, ***P < 0.001.

Furthermore, to further investigate the regulatory role of MGAT5 in DN, the effects of MGAT5 overexpression on oxidative stress and ferroptosis in DN cells were examined. Flow cytometry results showed that HG treatment increased the levels of ROS and lipid ROS, while MGAT5 overexpression significantly reduced ROS and lipid ROS levels under HG stimulation, whereas MGAT5 knockdown exerted the opposite effects (Figure 4A and B). Meanwhile, HG treatment increased MDA levels and decreased GSH levels, whereas MGAT5 overexpression reduced MDA levels, increased GSH levels, and decreased Fe^2+^ content; in contrast, MGAT5 knockdown produced the opposite effects (Figure 4C and D). Additionally, the assay results demonstrated that HG treatment increased Fe^2+^ levels. Under HG-induced conditions, MGAT5 overexpression significantly decreased Fe^2+^ levels, whereas MGAT5 knockdown resulted in the opposite effect (Figure 4E). Western blot analysis further revealed that HG treatment decreased GPX4 expression and increased the expression levels of ACSL4 and PTGS2. Under HG conditions, MGAT5 overexpression upregulated GPX4 expression while suppressing ACSL4 and PTGS2 expression, whereas MGAT5 knockdown exhibited the opposite trends (Figure 4F). These findings indicate that MGAT5 exerts a protective effect in HG-induced HPC cells by suppressing oxidative stress and regulating ferroptosis-related markers.

### 3.5 MGAT5 overexpression inhibits apoptosis and inflammation in HPC cells via activation of the Nrf2/HO-1 pathway

**Figure 5:**
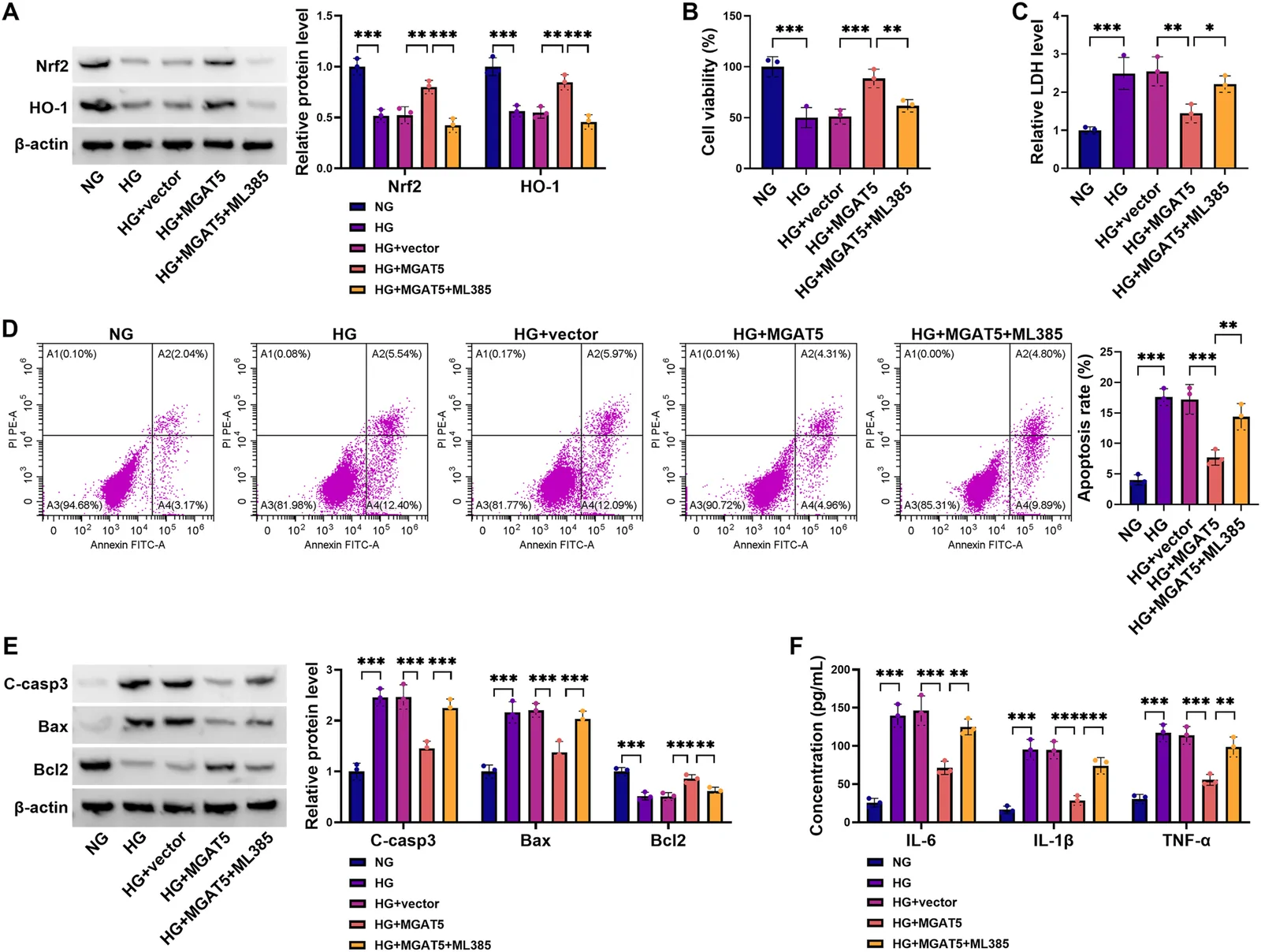
Regulatory effects of MGAT5 on the Nrf2/HO-1 pathway. (A) Effects of MGAT5 overexpression combined with ML385 on Nrf2 and HO-1 protein expression in DN models based on WB detection (*n* = 3). (B) Effects of MGAT5 overexpression combined with ML385 on cell viability in DN models based on CCK-8 detection (*n* = 3). (C) Effects of MGAT5 overexpression combined with ML385 on LDH levels in DN models based on colorimetric assay (*n* = 3). (D) Effects of MGAT5 overexpression combined with ML385 on apoptosis in DN models based on flow cytometry (*n* = 3). (E) Effects of MGAT5 overexpression combined with ML385 on protein expression of C-caspase3, Bax, and Bcl2 in DN models based on WB detection (*n* = 3). (F) Effects of MGAT5 overexpression combined with ML385 on IL-6, IL-1β, and TNF-α levels in DN models based on ELISA (*n* = 3). *P < 0.05, **P < 0.01, ***P < 0.001.

To further investigate the interaction between MGAT5 and the Nrf2/HO-1 pathway and their regulatory effects on DN, this study examined the effects of MGAT5 overexpression on Nrf2 and HO-1 proteins and evaluated their impacts on the biological functions of DN cell models. Under HG induction, MGAT5 overexpression significantly upregulated Nrf2 and HO-1 proteins, but this activation was blocked by the addition of the Nrf2 inhibitor ML385 (Figure 5A). Functional assays revealed that MGAT5 overexpression enhanced cell viability and reduced LDH release and apoptosis, effects that were reversed upon ML385 treatment (Figure 5B–D). WB analysis further showed that MGAT5 overexpression decreased the levels of C-casp3 and Bax while increasing Bcl2 expression, but these effects were partially restored in the presence of ML385 (Figure 5E). Moreover, MGAT5 overexpression significantly reduced IL-6, IL-1β, and TNF-α levels, and these anti-inflammatory effects were also reversed by ML385 (Figure 5F). These results indicate that MGAT5 plays a protective role in HG-induced HPC cells by activating the Nrf2/HO-1 pathway, and its regulation of cell viability, apoptosis, and inflammation depends on this pathway.

### 3.6 MGAT5 overexpression suppresses oxidative stress and ferroptosis in HPC cells through the Nrf2/HO-1 pathway

**Figure 6:**
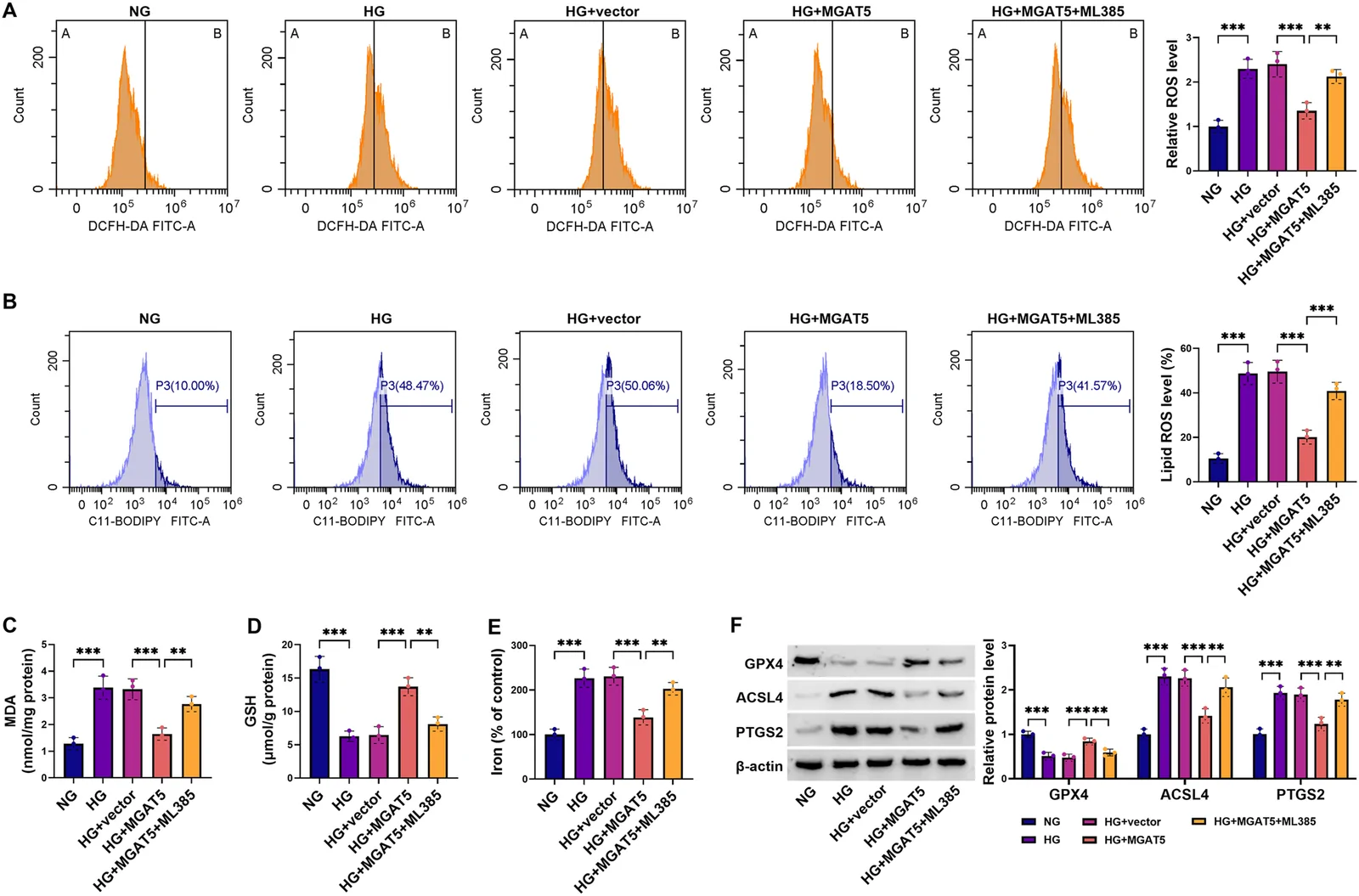
Effects of MGAT5 on oxidative stress and ferroptosis in DN cell models via the Nrf2/HO-1 pathway. (A) Effects of MGAT5 overexpression combined with ML385 on ROS levels in DN cell models measured by flow cytometry (*n* = 3). (B) The effect of MGAT5 overexpression combined with ML385 treatment on lipid ROS levels in the DN model was assessed by flow cytometry (*n* = 3). (C) Effects of MGAT5 overexpression combined with ML385 on MDA levels in DN cell models measured by colorimetric assay (*n* = 3). (D) Effects of MGAT5 overexpression combined with ML385 on GSH levels in DN cell models measured by colorimetric assay (*n* = 3). (E) Effects of MGAT5 overexpression combined with ML385 on Fe^2+^ levels in DN cell models measured by colorimetric assay (*n* = 3). (F) Effects of MGAT5 overexpression combined with ML385 on protein expression levels of GPX4, ACSL4, and PTGS2 in DN cell models measured by WB (*n* = 3). **P < 0.01, ***P < 0.001.

Based on the above findings, the effects of MGAT5 overexpression on oxidative stress and ferroptosis in DN cells were further investigated. Flow cytometry results showed that HG treatment increased ROS and lipid ROS levels. Under HG stimulation, MGAT5 overexpression significantly reduced cellular ROS and lipid ROS levels, whereas MGAT5 overexpression combined with ML385 treatment produced the opposite effects (Figure 6A and B). Meanwhile, HG treatment increased MDA levels and decreased GSH levels, while MGAT5 overexpression reduced MDA levels, increased GSH levels, and decreased Fe^2+^ content. However, MGAT5 overexpression combined with ML385 treatment exerted opposite effects (Figure 6C and D). In addition, the kit-based assay showed that HG treatment increased Fe^2+^ levels. Under HG conditions, MGAT5 overexpression significantly reduced Fe^2+^ levels, whereas MGAT5 overexpression combined with ML385 treatment resulted in the opposite effect (Figure 6E). Western blot analysis further demonstrated that HG treatment decreased GPX4 expression and increased ACSL4 and PTGS2 expression. Under HG conditions, MGAT5 overexpression increased GPX4 expression while suppressing ACSL4 and PTGS2 expression, whereas MGAT5 overexpression combined with ML385 treatment showed the opposite trend (Figure 6F). These findings indicate that MGAT5 may alleviate HG-induced oxidative stress and ferroptosis through a mechanism involving the Nrf2 signaling pathway, thereby exerting a protective effect on DN cells.

### 3.7 MGAT5 regulates DN cell phenotype and ferroptosis through the Nrf2/HO-1 signaling pathway

Based on the above findings, further investigation was conducted to explore the regulatory effects of MGAT5 and Nrf2 on the phenotype, oxidative stress, and ferroptosis of DN cell models. First, western blot analysis showed that Nrf2 knockdown significantly reduced Nrf2 protein expression, indicating the successful knockdown of Nrf2 (Figure S1A). CCK-8 and flow cytometry analyses demonstrated that HG stimulation significantly decreased cell viability and increased the apoptosis rate. Under HG conditions, MGAT5 overexpression significantly increased cell viability and reduced apoptosis, whereas MGAT5 overexpression combined with Nrf2 knockdown resulted in decreased cell viability and increased apoptosis (Figure S1B–C). Meanwhile, ELISA, flow cytometry, and kit-based assays showed that HG treatment significantly increased the levels of IL-6, IL-1β, and TNF-α, as well as lipid ROS and Fe^2+^ levels. Under HG conditions, MGAT5 overexpression significantly decreased the levels of IL-6, IL-1β, and TNF-α, along with reduced lipid ROS and Fe^2+^ levels, whereas MGAT5 overexpression combined with Nrf2 knockdown produced the opposite effects (Figure S1D–F). Collectively, these findings suggest that MGAT5 may participate in the regulation of HG-induced DN cell phenotypic changes, inflammatory responses, oxidative stress, and ferroptosis through a mechanism dependent on the Nrf2/HO-1 signaling pathway.

## 4 Discussion

Hyperglycemia is the major cause of diabetes [38], and DN is one of the most common complications of diabetes mellitus [39]. Under hyperglycemic conditions, multiple pathological mechanisms, including metabolic disorder, oxidative stress, inflammatory response, apoptosis, and fibrosis, interacted and eventually led to renal structural and functional impairment, thereby accelerating the progression of DN [40], [41], [42], [43], [44]. Recent evidence further indicates that modulation of antioxidant and inflammatory signaling pathways, particularly the Nrf2-related network, represents an important mechanism underlying renal protection in kidney injury models [45]. Therefore, this study establishes an *in vitro* DN model by exposing HPC cells to HG to explore the underlying mechanisms. Previous research has indicated that the Nrf2/HO-1 pathway is critically involved in safeguarding against DN progression [46], [47], [48]. However, the upstream and downstream regulatory mechanisms of this pathway remain unclear, and it remains uninvestigated whether MGAT5 exerts its function through this pathway. In addition, MGAT5 was a key N-acetylglucosaminyltransferase that participated in the complex N-glycosylation of glycoprotein [49], 50]. Studies also indicated that MGAT5 affected processes such as cell adhesion, signal transduction, and inflammatory response [51], [52], [53]. The development of DN was closely associated with processes such as hyperglycemia-induced oxidative stress and inflammatory response [54]. Therefore, this study focuses on the regulatory mechanism of MGAT5 in DN; however, no study has confirmed the regulatory role of MGAT5 in DN, and this study aims to fill this gap. MGAT5 is involved in the development of multiple diseases, where it may exert distinct regulatory functions depending on the disease context [55], 56]. The present study found that MGAT5 expression was downregulated in the renal tissues of DN patients, suggesting that it might play a protective role in DN.

In terms of mechanism, this study systematically investigates the influence of MGAT5 on a range of cellular injury markers. LDH, an enzyme involved in glycolysis and lactate metabolism, is released upon membrane damage or necrosis and serves as an indicator of cellular injury [57]. Regarding apoptosis-related proteins, C-casp3 and Bax promote cell death, while Bcl2 counteracts apoptosis by inhibiting Bax channel formation and preventing cytochrome C release [58], 59]. IL-6 and IL-1β serve as strong pro-inflammatory cytokines that enhance inflammatory responses [60], 61], whereas TNF-α not only induces apoptosis but also promotes the secretion of additional inflammatory mediators [62]. Recent studies have also highlighted the interaction between antioxidant pathways and inflammatory signaling, indicating that activation of Nrf2 can simultaneously suppress oxidative damage and inflammatory responses [63]. As for oxidative stress, excessive accumulation of ROS and MDA caused oxidative damage [64], 65], while GSH functioned as a critical antioxidant to scavenge free radicals and preserve cellular integrity [66]. Consistent with previous studies demonstrating that activation of antioxidant pathways such as Nrf2 signaling can alleviate renal injury by suppressing oxidative stress, inflammation, and apoptosis, these findings further suggest that MGAT5 may contribute to renal protection through regulation of this antioxidant defense system [67]. Iron metabolism and ferroptosis were also implicated: Fe^2+^ overload exacerbated cell death [68], GPX4 acted as a key suppressor of ferroptosis by clearing lipid peroxides via GSH [69], ACSL4 facilitated lipid peroxidation of polyunsaturated fatty acids [70], and PTGS2 was regarded as a marker of both inflammation and ferroptosis [71]. In this study, MGAT5 overexpression significantly reduced the concentrations of LDH, IL-6, IL-1β, TNF-α, ROS, MDA, Fe^2+^, ACSL4, and PTGS2, reduced apoptosis rates as well as C-casp3 and Bax expression, while enhancing the levels of Bcl2, GSH, and GPX4, indicating that MGAT5 exerts multifaceted protective effects against HG-induced injury in HPC cells.

This study further verified the regulatory role of MGAT5 on the Nrf2/HO-1 pathway using ML385, a specific inhibitor of Nrf2. The results showed that the protective effects of MGAT5 on cell viability, programmed cell death, immune response, and reactive oxygen species all relied on the activation of the Nrf2/HO-1 pathway. Previous studies have similarly shown that Nrf2/HO-1 signaling alleviates DN progression by modulating oxidative stress, inflammatory responses, and apoptosis [24], 46]. However, MGAT5 is identified for the first time as an upstream regulator of the Nrf2/HO-1 pathway, which constitutes the major novelty of this study.

However, this study has several limitations. The identification of MGAT5 was mainly based on bioinformatics analysis, and further validation using independent datasets or clinical samples is required. In addition, the current findings were obtained primarily from an HG-induced HPC cell model, and therefore should be interpreted cautiously regarding the complex pathogenesis of DN. Moreover, the lack of *in vivo* validation using animal models or human samples limits the further confirmation of the clinical relevance of our findings. Furthermore, although the results suggest that MGAT5 may regulate the Nrf2/HO-1 signaling pathway, the detailed molecular mechanisms underlying this regulation remain unclear. The current study did not investigate whether MGAT5 directly affects Nrf2 activation through glycosylation modification, protein–protein interactions, or the Keap1-Nrf2 axis. Moreover, the partial reversal of MGAT5-mediated protective effects following Nrf2/HO-1 inhibition suggests that other Nrf2/HO-1-independent mechanisms may also be involved, which require further investigation. Further studies involving mechanistic approaches, such as co-immunoprecipitation, glycosylation analysis, and Nrf2 nuclear translocation assays, are warranted to elucidate the precise regulatory mechanism of MGAT5 in DN progression.

In summary, this study finds that MGAT5 expression is reduced in DN patients and shows that MGAT5 alleviates HG-induced damage in HPC cells by triggering the Nrf2/HO-1 signaling cascade, thereby providing antioxidant, anti-inflammatory, anti-apoptotic, and anti-ferroptosis protection. This highlights MGAT5 as a key factor in the pathogenesis of DN and provides a potential therapeutic target and theoretical basis for its prevention and treatment. Future research may further explore the role of MGAT5 in animal models and clinical samples, offering new perspectives for precision therapy in DN.

## Supplementary Material

Supplementary Material

Supplementary Material
