# Supplementary material for: MGAT5 regulates ferroptosis via the Nrf2/HO-1 signaling pathway in diabetic nephropathy
Source: Open Life Sci. 2026 Sep 8;21(1):20251362. doi: 10.1515/biol-2025-1362 (PMC13552620; doi:10.1515/biol-2025-1362)
Supplement: Supplementary file 1 [file j_biol-2025-1362_suppl_002.docx]

**Figure S1:** Effects of MGAT5 on the phenotype and ferroptosis of DN cell models via the Nrf2/HO-1 pathway.

(A) Western blot analysis of Nrf2 protein expression levels in the negative control group (siNC) and Nrf2 knockdown group (siNrf2) (n = 3). (B) CCK-8 assay of cell viability in normal glucose (NG), high glucose (HG), high glucose combined with MGAT5 overexpression (HG+MGAT5), high glucose combined with MGAT5 overexpression and negative control group (HG+MGAT5+siNC), and high glucose combined with MGAT5 overexpression and Nrf2 knockdown group (HG+MGAT5+siNrf2) (n = 3). (C) Flow cytometry analysis of cell apoptosis levels in normal glucose (NG), high glucose (HG), high glucose combined with MGAT5 overexpression (HG+MGAT5), high glucose combined with MGAT5 overexpression and negative control group (HG+MGAT5+siNC), and high glucose combined with MGAT5 overexpression and Nrf2 knockdown group (HG+MGAT5+siNrf2) (n = 3). (D) ELISA analysis of IL-6, IL-1β, and TNF-α levels in normal glucose (NG), high glucose (HG), high glucose combined with MGAT5 overexpression (HG+MGAT5), high glucose combined with MGAT5 overexpression and negative control group (HG+MGAT5+siNC), and high glucose combined with MGAT5 overexpression and Nrf2 knockdown group (HG+MGAT5+siNrf2) (n = 3). (E) Flow cytometry analysis of lipid ROS levels in normal glucose (NG), high glucose (HG), high glucose combined with MGAT5 overexpression (HG+MGAT5), high glucose combined with MGAT5 overexpression and negative control group (HG+MGAT5+siNC), and high glucose combined with MGAT5 overexpression and Nrf2 knockdown group (HG+MGAT5+siNrf2) (n = 3). (F) Kit-based determination of Fe^2+^ levels in normal glucose (NG), high glucose (HG), high glucose combined with MGAT5 overexpression (HG+MGAT5), high glucose combined with MGAT5 overexpression and negative control group (HG+MGAT5+siNC), and high glucose combined with MGAT5 overexpression and Nrf2 knockdown group (HG+MGAT5+siNrf2) (n = 3). ***P* < 0.01, ****P* < 0.001.
